# Evaluation of barbed suture for celiorrhaphy and subcutaneous closure in bitches with pyometra submitted to ovariohysterectomy

**DOI:** 10.1590/ACB360502

**Published:** 2021-06-14

**Authors:** Helen Cristina Gomes de Lima, Alexandre Pinto Ribeiro, Jéssica Ávila de Souza, Raphael Rogger Vieira, Michelly Ferreira Fernandes

**Affiliations:** 1 Resident. School of Veterinary Medicine - Universidade Federal de Mato Grosso - Cuiaba (MT), Brazil.; 2 PhD, Associate Professor. School of Veterinary Medicine - Universidade Federal de Mato Grosso - Cuiaba (MT), Brazil.; 3 Resident. School of Veterinary Medicine - Universidade Federal de Mato Grosso - Cuiaba (MT), Brazil.

**Keywords:** Surgical Time, Abdominal Wall, Ultrasonography

## Abstract

**Purpose:**

To evaluate the use of barbed sutures over the surgical time, the leukogram, the tissue thickness in which the sutures were employed (ultrasonography), the costs, and the possible complications in bitches with pyometra submitted to ovariohysterectomy (OH).

**Methods:**

Convectional 2.0 polyglyconate suture was used in the control group (CG n = 10) and 2.0 barbed polyglyconate suture in the barbed group (BG n = 10) to perform celiorrhaphy (simple continuous pattern) and subcutaneous closure (continuous intradermal pattern). Data were assessed using paired (leukogram between 24 and 48 h within the same group) and unpaired (leukogram, surgical time, tissue thickness, and costs) Student’s t-test. The Fisher exact test was used to assess the occurrence of seroma between groups (p < 0.05). Results are shown as mean ± standard error of mean.

**Results:**

The time spent to perform the celiorrhaphy (195.30 ± 17.37 s vs. 204 ± 16.00 s), subcutaneous closure (174.0 ± 15.86 s vs. 198.0 ± 15.62 s), and the total surgical time (24.30 ± 1.44 min vs. 23.00 ± 1.30 min) did not differ between BG and CG, respectively (p > 0.05). Leukogram at 48 h post-surgery did not differ between groups (p = 0.20). No differences were observed in the subcutaneous and the abdominal wall thickness (cm) assessed by ultrasonography at 48 h in BG (0.31 ± 0.04, 0.80 ± 0.05) and CG (0.34 ± 0.03, 0.72 ± 0.06), respectively. Similarly, 15 days post-surgery the same structures did not differ between BG (0.26 ± 0.02, 0.74 ± 0.08) and CG (0.26 ± 0.03, 0.64 ± 0.05) (p > 0.05). In one bitch from each group, a mild seroma was observed on one side of the surgical wound 48 h after surgery (p = 1.00). The procedures in which barbed sutures were used had an average additional cost of R$ 200.00 ± 11.66 (p < 0.0001).

**Conclusions:**

Barbed suture has proven to be efficient and safe for abdominal and subcutaneous closure. However, considering its current high cost in addition thatthe surgical time of bitches with pyometra undergone OH was not reduced, no advantages were observed with theuse of barbed sutures for this type of surgery.

## Introduction

Canine pyometra is a condition characterized by chronic inflammation of the endometrium that results in bacterial colonization, systemic inflammatory response and sepsis, with ovariohysterectomy (OH) being the most effective treatment^1^. Although OH is a routine procedure, anesthetizing bitches suffering from severe systemic disease and/or organ malfunctions may be hazardous^1^. Therefore, techniques aimed at reducing both anesthesia and surgery times may increase the probability of achieving satisfactory outcomes.

Recently, sutures with unidirectional barbs have been developed. These barbs allow the suture to pass through the tissue in one direction without undue friction, and to create an anchor within the tissue, allowing for a more even distribution of tension along wound edges. In addition, these sutures eliminate the need for a terminal knot, resulting in a possible decrease in surgical time^2^. Cadaveric studies conducted in dogs have examined the use of barbed sutures in enterotomy, cystotomy, frenotomy, intradermal, and fascia lata closures^2^–^6^. Some experiments have reported that the use of barbed sutures significantly reduced the surgical time in open abdominal procedures in dogs^3^,^7^.

*In vivo* and *ex vivo* studies showed that barbed sutures have a significantly lower maximum load at failure, decreased stiffness, and higher average tissue reaction scores when used for the closure of skin incisions in dogs,in comparison with conventional monofilament sutures^8^,^9^. Inveterinary medicine, there are no reports describing the use of barbed sutures for celiotomy closure in open procedures. Experimental enterorrhaphies developed in cadavers of horses had higher costs in constructs made with barded sutures10. In the clinical setting, however, studies evaluating if the higher cost of barbed sutures are able to decrease the final cost spent with the hospital bills have never been published in the veterinary literature. Considering the possible advantages in reducing the surgical time along with the possible abnormalities resulting from the spurs displaced in both subcutaneous and muscular tissues, it seems reasonable to investigate the use of barbed sutures in clinical cases of pyometra^1^,^8^. Thus, the objectives of the present study are to evaluate and compare the surgical time, the subcutaneous and *linea alba* thickness, the leukogram changes, the cost, and the possible postoperative complications in bitches affected by pyometra, undergoing midline celiotomy and subcutaneous closure with barbed or conventional monofilament sutures.

## Methods

### Animals, procedures and groups

All procedures were approved by the Institutional Committee for Ethics in the Use of Animals, on September 01, 2017 (protocol No. 23108. 944050/2018-35).

Bitches with leukocytosis and an ultrasonographic diagnosis of enlarged uterine horns, with or without fever, that underwent OH were pre-selected to be enrolled in the study. Before surgery all animals received intramuscular methadone (mg·kg^–1^). General anesthesia was induced with intravenous (IV) propofol (10 mg·kg^–1^ as needed), maintained with inhaled isoflurane in 100% oxygen, and monitored throughout. All bitches received epidural anesthesia with lidocaine (4.5 mg·kg^–1^) not exceeding a total volume of 5 mL. At the end of surgery, each patient was medicated with IV meloxicam (0.2 mg·kg^–1^).

Open OH was performed with a retro-umbilical midline approach. In order to avoid disparities, the skin incision was always equal in size to the incision performed in the *linea alba*. Thus, as inclusion criteria, only bitches weighing between 5 and 10 kg were recruited. In all animals, OHs were performed with hemostatic forceps and ligation of the ovarian and uterine vessels with 2.0 polyglactin 910. For abdominal and subcutaneous closure, bitches were randomly assigned (http://www.jerrydallal.com/random/randomize.htm) in two different groups (n = 10 each). In the barbed group (BG), the celiotomy and the subcutaneous tissue were closed with 2.0 barbed polyglyconate (V-LOC 180-COVIDIEN). In the conventional group (CG), the incisions were closed using conventional 2.0 polyglyconate sutures without barbs (Maxon-COVIDIEN). In both groups, the celiotomy was closed with a simple continuous pattern, whereas the subcutaneous tissue was closed with a continuous intradermal pattern tacking the suture in the deep abdominal fascia. For skin incision closure, a simple interrupted pattern with 2.0 nylon sutures was used in all cases. After OH, hysterocentesis was performed on each uterus and the samples collected were placed in a sterile container to assess for bacterial growth and confirm the diagnosis of pyometra.

**Figure 1:**
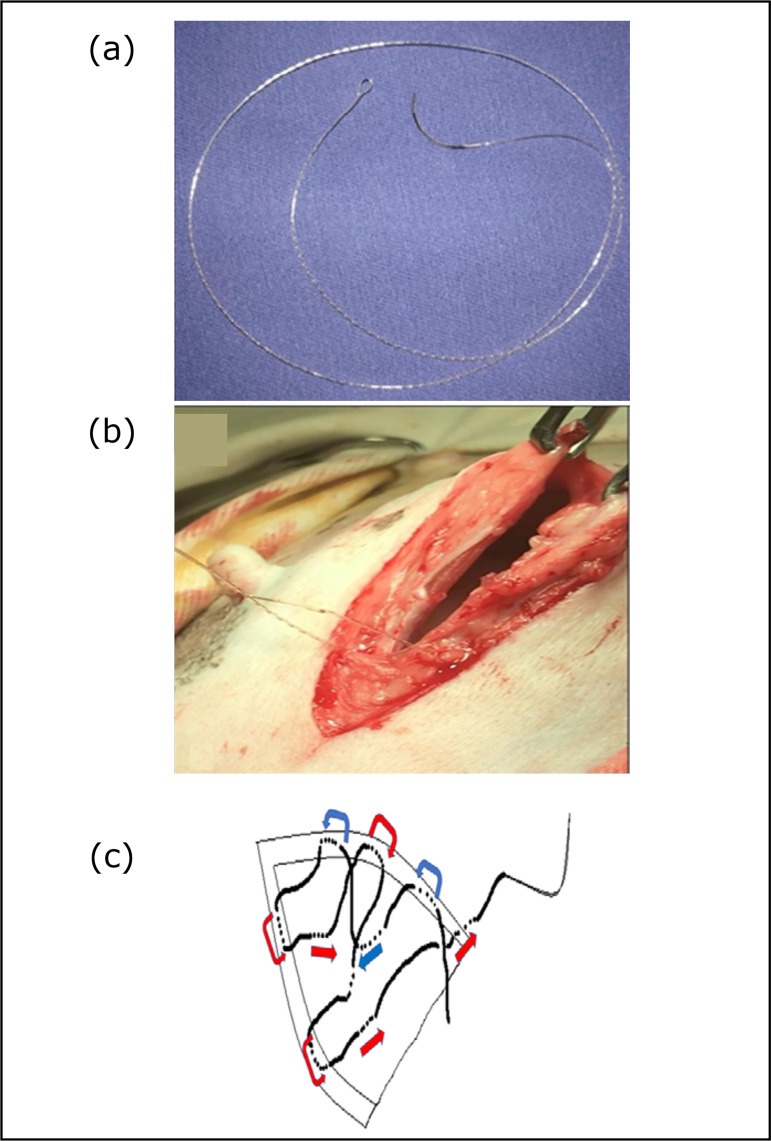
**(a)** Barbed suture with anchor loop at eh extremity; **(b) I**nitial suture anchor loop was formed by passing the needle through the unidirectional strand loop; **(c)** Two-needle end-pass technique was used initiate and finish the suture line.

To accomplish the celiotomy closure knot in the animals of BG, the initial suture anchor loop was formed by passing the needle through the unidirectional strand loop (Fig. 1a–b). In this group, each suture line was completed with a two-needle end-pass technique, as described in the veterinary literature (Fig. 1c)^2^. Because the same barbed suture material was used for subcutaneous tissue closure and once the anchor loop was formed, the standard technique described when barbed sutures are used had to be modified. Therefore, the initial sutures performed in the subcutaneous closure commenced with a two-needle start-pass technique from the left to the right (caudally). Thereafter, the pattern described previously to close this layer was performed from the right to the left (cranially), and the suture line was terminated with a two-needle end-pass technique. The length of the incisions, the time to accomplish the celiotomy, the subcutaneous closures, and the total surgical time were assessed for both groups.

### Ultrasonographic evaluations and post-operative care

In the first 24 h, 48 h and 10 days post-surgery, the incidence of seroma at the surgical wound was assessed with ultrasonography. Hematological and basic serum biochemistry profiles were obtained in all cases 24 h pre-surgery and 48 h post-surgery. The ultrasonographic evaluation was performed with a 12 MHz transducer, and the average of three longitudinal cuts were used to assess the *linea alba* and the subcutaneous thickness (cm). The assessment of *linea alba* and the subcutaneous thickness were performed 48 h and 15 days post-surgery. In the first 72 h of the postoperative period, IV lactated Ringer’s solution was administered (10 mL·kg^–1^). During this period, bitches were also treated with IV ceftriaxone (25 mg·kg^–1^) every 12 h, subcutaneous (SC) tramadol (3 mg·kg^–1^) every 6 h, and SC meloxicam (0.2 mg·kg^–1^) every 24 h. Surgical wounds were cleansed every 12 h with 0.2% chlorhexidine solution. After this period patients were discharged when urinary protein-to-creatinine ratio was less than 1.7 mg·dL^–1^. Oral antibiotic was prescribed based on culture and sensitivity test results of the uterine contents. At the recheck 10 days after surgery, the sutures were removed.

### Statistical analysis

Data were tested for normal distribution using the Shapiro–Wilk test. Comparisons between the groups regarding the final cost, the results of the hematological and biochemical profiles, the times to perform the celiotomy and the subcutaneous closures, as well as the subcutaneous and *linea alba* thickness evaluated by ultrasonography, were assessed with an unpaired Student’s t-test. Results of the hematological and biochemical profiles obtained between 24 and 48 h after surgery were compared within patients of the same group with a paired Student’s t-test. The Fisher exact test was used to assess the occurrence of seroma between groups. Significance was set at p < 0.05. Results are shown as mean ± standard error of mean (SEM).

## Results

### Clinical data and surgical times between groups

Mean ± SEM age in years was 7.7 ± 0.91 and 8.7 ± 1.3 in the BG and CG patients, respectively (p = 0.53). Mean ± SEM weight in kg was 9.68 ± 1.06 and 9.94 ± 1.2 in the BG and CG patients, respectively (p = 0.87). In both groups, the preoperative values of creatinine, alanine aminotransferase, platelet count, and total protein where within the reference range for dogs, remaining the same 48 h after surgery (p > 0.05). During the first 24 and 48 h post-surgery, blood counts of the patients in both groups revealed regenerative anemia and leukocytosis (p > 0.05) (Table 1). Bacterial growth from the uterine contents was confirmed in all patients. From the BG patients, seven tested positive for *Escherichia coli*, one for *Streptococcus* spp., and two for *Citrobacter* spp. From the CG patients, eight tested positive for *E. coli* and two for *Streptococcus* spp. All cultures were sensitive to amoxicillin. Surgical length incisions did not differ between the groups (p = 0.95). Likewise, the time taken to perform the celiotomy (p = 0.71) and the subcutaneous closures (p = 0.29), as well as the total surgical time did not differ between the groups (p = 0.51) (Table 2).

**Table 1:** Mean ± standard error and probability (P) results of the hematological and biochemical profiles before surgery and 48 h post-surgery in barbed (BG) and control groups (CG).

Variables	Before surgery				48 h post-surgery		
	BG	CG	P		BG	CG	P
Hematocrit (%)	32.34 ± 2.82	39.34 ± 2.37	0.08		31.95 ± 2.16	35.11 ± 2.46	0.34
Erythrocytes (x 10^6^·uL^–1^)	5.36 ± 0.17	5.85 ± 0.20	0.08		5.16 ± 0.16	5.69 ± 0.22	0.07
Leukocytes (x 10^3^·uL^–1^)	33.06 ± 6.22	27.62 ± 3.16	0.44		50.40 ± 8.58	37.34 ± 4.88	0.20
Platelets (x 10^3^·µL^–1^)	275.30 ± 15.22	317.60 ± 26.10	0.17		287.40 ± 16.80	286.90 ± 25.90	0.98
Total protein (g·dL^–1^)	9.64 ± 0.38	9.80 ± 0.54	0.81		9.22 ± 0.45	9.14 ± 0.5٩	0.91
Albumin (g·dL^–1^)	2.27 ± 0.19	2.50 ± 0.15	0.36		2.23 ± 0.1٩	2.26 ± 0.1٩	0.91
Creatinine (mg·dL^–1^)	0.81 ± 0.00	1.00 ± 0.09	0.18		0.80 ± 0.10	1.05 ± 0.10	0.11
Alanine aminotransferase (UI·L^–1^)	20.60 ± 3.18	37.00 ± 7.71	0.06		23.80 ± 3.12	34.20 ± 4.19	0.06

**Table 2:** Mean ± standard error (minimum and maximum) and probability (P) of the length of the incisions, the times to perform the celiorrhaphy, the subcutaneous tissue closures, and the total surgical time in barbed and control groups.

Variables	Barbed group	Control group	P
Incision length (cm)	5.71 ± 0.32 (4.3–8.0)	5.67 ± 0.27 (5.0–7.0)	0.92
Celiorrhaphy (s)	195.30 ± 17.37 (120–300)	204.00 ± 16.00 (120–300)	0.71
Subcutaneous closure (s)	174.00 ± 15.86 (120–240)	198.00 ± 15.62 (120–300)	0.29
Total surgical time (min)	24.30 ± 1.44 (15–30)	23.00 ± 1.30 (18–30)	0.51

### Ultrasonographic evaluations and final cost

In one bitch from each group, a mild seroma was observed on one side of the surgical wound 48 h after surgery (p = 1.00), which was treated with a needle aspiration. The ultrasonograms performed at 48 h and 15 days post-surgery did not reveal statistical differences in the subcutaneous tissue and the abdominal wall thickness values between groups (p > 0.05) (Table 3). Ten days after surgery, the sutures were removed. At this recheck, the surgical wounds had healed in all study patients without signs of suture dehiscence, seroma, inflammation, or bleeding.

**Table 3:** Mean ± standard error (minimum and maximum) and probability (P) of the ultrasonographic measurements of the subcutaneous and *linea alba* thickness obtained 48 h and 15 days after surgery in barbed and control groups.

Variables		Barbed group	Control group	P
*Linea Alba*				
	48 h	0.80 ± 0.05 (0.55–1.08)	0.72 ± 0.06 (0.39–1.08)	0.38
	Day 15	0.74 ± 0.08 (0.17–1.16)	0.64 ± 0.05 (0.33–0.90)	0.34
Subcutaneous tissue				
	48 h	0.31 ± 0.04 (0.14–0.56)	0.34 ± 0.03 (0.23–0.56)	0.58
	Day 15	0.26 ± 0.02 (0.17–0.45)	0.26 ± 0.03 (0.15–0.48)	0.92

The final cost of each OH performed with barbed and conventional polyglyconate sutures was R$ 885.50 ± 8.25 and 685.50 ± 8.25, respectively. The procedures in which barbed sutures were used had an average additional cost of R$ 200.00 ± 11.66 (p < 0.0001).

## Discussion

The present study is the first to report the use of barbed suture to perform celiorrhaphy and subcutaneous closure in patients with pyometra who have undergone open OH. Bitches affected by pyometra are usually presented with related hematological and serum biochemical imbalances^1^. In order to avoid bias, the aim was to select patients in which the results of such laboratory findings did not show statistical differences between groups. As expected, 48 h after surgery increased leukocyte counts were observed in both groups. During this period, this finding could be associated with the systemic effects of the infection itself, as well as the surgical manipulation, before an effective antibiotic therapy was instituted based on bacterial cultures. The prescription of anti-inflammatory drugs in both groups is another factor that may have skewed the interpretation of leukograms within and between the groups. Therefore, the results of leukocyte counts could not be considered a reliable tool to predict if barbed sutures were able to achieve an inflammatory reaction in the study population.

Ultrasonography is considered to be an adequate method to subjectively evaluate the degree of inflammation in muscles and tendons^11^–^13^. In this study, the thickness values of the muscles that form the *linea alba* and the subcutaneous tissue did not differ significantly between the groups. These findings might contrast with previous results reported in the literature^8^,^14^. Regarding the inflammatory content, one study developed in rats reveled that the celiorrhaphy area repaired with conventional polypropylene showed higher macrophage infiltration 7 days after surgery^15^. Another study reported increased macrophage infiltration in the *linea alba* of rabbits that underwent experimental celiorrhaphy with barbed sutures, three weeks after the procedures^14^ . Additionally, an *in vivo* experimental study conducted in dogs has demonstrated higher inflammatory reaction scores in individuals in which skin incisions were closed with barbed sutures, in comparison with those closed with conventional sutures^8^. Despite other studies having suggested that barbed sutures might be more traumatic than conventional sutures of the same material when performing celiorrhaphy and subcutaneous closures, the ultrasonographic evaluations could not support this finding. The histological evaluation of muscles in the *linea alba* and skin biopsies to assess abnormalities regarding cellular infiltrate, hemorrhage, necrosis, and fibrosis, along with quantitative analysis of inflammatory cytokines would be more elucidative than the parameters used here, but the clinical nature of this study did not allow such an investigation.

The present study showed that the use of knotless barbed sutures did not promote a reduction in total surgical time, or in the time needed to perform celiorrhaphy and subcutaneous closures in dogs. Results of veterinary studies that have evaluated the time necessary to accomplish subcutaneous closures in dogs are controversial^8^,^16^. One experimental study has shown reduced surgical times when barbed sutures were used^8^, whereas in another study conducted in clinical cases, there was no statistical difference in surgical time between barbed and conventional sutures16. These results point to the fact that an absence of statistical difference in the surgical times between the two groups can be related to the ease and speed with which surgical knots are accomplished in open abdominal surgeries when a continuous pattern is used. However, this case series was conducted by one single surgeon with expertise in performing celiorrhaphy with conventional sutures. Further studies should evaluate if the use of barbed sutures could have an impact in the surgical time of celiorrhaphy performed by multiple residents of surgery at the early learning curve.

Studies in some animal species have investigated a possible correlation between a higher biomechanical stress on the muscular fascia and the spurs present in the barbed sutures^5^,^14^,^17^. In one study, no additional advantage was reported regarding the collagen deposition in the surgical wound in rabbits, that underwent celiorrhaphy when comparing conventional versus barbed polydioxanone sutures (PDS)^14^. Additionally, authors of the same study described that the maximum load at failure did not differ between groups^14^. Another *in vivo* study reported similar results in pigs that underwent laparotomy closures with barbed or smooth PDS^17^. One study, however, conducted on the fascia lata of dog cadavers, showed a lower maximum load at failure in constructs made with barbed PDS than those made with similar suture material without barbs^5^. Results of *in vivo* and *ex vivo* canine studies have also shown that the maximum load at failure did not differ when barbed or smooth sutures were used for intradermal skin closures^8^,^9^. The clinical nature of this study did not allow the use of biomechanical tests for such an investigation. Another failure of the present study was not being possible to compare the occurrence visceral adhesions in the abdominal wall of both groups, once different materials may induce such abnormality^18^.

In humans, one clinical study reported that performing subcutaneous closure with standard absorbable sutures in a continuous pattern resulted in a greater incidence of complications (infections, wound problems, and seromas) than did closures completed using barbed sutures^19^. On the contrary, experimental studies conducted in animals described short-term outcomes, with no adverse events (incisional hernia in pigs or wound dehiscence in dogs) that have received either conventional or barbed sutures^8^,^17^. Likewise, incisional hernia and wound dehiscence were not seen in this study, suggesting that 2.0 polyglyconate barbed sutures are able to sustain an adequate tensile strength for the abdomen and subcutaneous tissue in dogs with an average weight of 9.5 kg. It is important to mention that the cutting spurs present in a monofilament barbed suture decreases its effective diameter, resulting in a suture strength that is similar to that for a smooth monofilament suture one size smaller^9^. The presence of seroma observed 48 h post-surgery in one bitch of each group cannot be associated with the type of material and the diameter of the sutures used. Thus, the suggestion is that further studies enrolling a larger sample size, as well as patients suffering from other clinical conditions, are necessary to conclude whether barbed sutures provide additional benefits over conventional sutures, particularly regarding seroma formation.

Despite barbed sutures being more expensive than its conventional counterparts^10^, three studies have shown that the use of barbed sutures reduced the time in the operating room in patients who have undergone spinal surgery^20^ and total knee arthroplasty^21^,^22^. The same studies reported that the reduction in the time spentin the operating room was associated with a decrease inthe final cost of the procedures. In the present study, a single piece of barbed 2.0 polyglyconate was used for suturing both the abdominal wall and the subcutaneous tissue; these procedures had no reduction in the total surgical time and an additional cost of R$ 200.00. Therefore, the popularization of barbed sutures among veterinary surgeons lies upon the reduction of the current cost along with clinical results, proving more benefits over conventional sutures.

## Conclusion

The use of barbed sutures has proven to be safe and effective for closing celiotomy and intradermal incisions in bitches with pyometra who have undergone open OH. The similar thickness values observed in the subcutaneous and *linea alba* of dogs of both groups suggest that the spurs present in the suture does not induce more tissue damage than conventional sutures. Considering the current higher costs and because they did not decrease the surgical time, there are currently no advantages for use barbed sutures for this type of procedure.
